# Docking Horses’ Tails

**Published:** 1893-04

**Authors:** 


### DOCKING HORSES' TAILS.

The Royal Agricultural Society of England has wisely proposed to give no prize to foals that have been docked, and when England gives up the fad our American dudes will abandon it as an English craze. An English writer explaining to tenant farmers the loss they sustain in docking their foals, says: "Having made inquiries of the great London dealers about high-class carriage horses, I find they will not buy them if docked, as they are made unsalable thereby. It is also a rule made by army remount purchasers that no horses are taken which have been docked." At the agricultural show lately held at Welbeck, where the Duke of Portland gave away a large sum for prizes for horses bred by his tenants, and which is worthy of all praise, he particularly requested his tenants to abstain from docking foals, as so injurious to their own interests; and at the autumnal show, at Elvasten Castle, of horses bred in the neighborhood, the same advice was given by Lord Harrington, both of these being large horse breeders.

